# Spectrophotometric Determination of Captopril and Penicillamine through the Use of Ligand Exchange Complexation Reactions

**Published:** 2011-12

**Authors:** Saied Belal, Omayma Abdel-Razak, Abdel-Fattah El-Walily, Rania Bakry

**Affiliations:** 1 *Department of Pharmaceutical Analytical Chemistry, Faculty of Pharmacy, University of Alexandria, Alexandria 21521, Egypt;*; 2 *Department of Pharmaceutical Chemistry, Faculty of Pharmacy, King Abdulaziz University, Jeddah, Saudi Arabia;*; 3 *Institute of Analytical Chemistry and Radiochemistry, Leopold Franzens University Innsbruck, Innrain 52a, 6020 Innsbruck, Austria*

**Keywords:** spectrophotometry, captopril, penicillamine, ligand exchange reactions

## Abstract

Two spectrophotmetric methods based on combined redox - ligand exchange reactions were developed for the determination of captopril and penicillamine in pure form and in their dosage forms. The first method is based on attenuating the absorbance of a ternary complex: silver (I) - bromopyrogallol red - phenanthroline in a buffer solution of pH6-8. The method has the concentration ranges 2-10 μg mL^-1^ and 0.5-1.75 μg mL^-1^ for captopril and penicillamine respectively, and the detection limits 7.1 × 10^-2^ and 5.7 × 10^-2^ μg mL^-1^ for captopril and penicillamine respectively. The second method is based on decreasing the absorbance of a chloroformic solution of copper (II) - oxine chelate when shaken with the drug solution in buffer medium of pH 8. The drugs were determined in the concentrations 30-90 μg mL^-1^ and 30-100 μg mL^-1^ for captopril and penicillamine respectively, and the detection limits 0.94 and 1.76 μg mL^-1^ for captopril and penicillamine respectively. The proposed methods were applied in the analysis of both compounds in their pharmaceutical preparations, and results were favorably compared with reference spectrophotometric methods regarding accuracy and precision.

## INTRODUCTION

**Figure 1:**
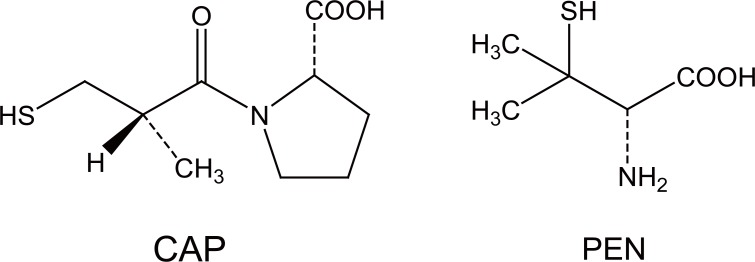
Chemical structures of captopril (CAP) and penicillamine (PEN).

Captopril (CAP) (Figure 1) is commonly used antihypertensive drug for which several methods have been described these methods including potentiometry (1), amperometry (2), stripping voltammetry (3, 4), fluorimetric (5), gas chromatographic (6, 7), and HPLC employing different detectors for its determination in biological fluids (8-10).

The reported spectrophotometric methods ranged from derivative and difference spectrophotometry (11-13), photometric measurement based on its reducing action (14-16), complex forming or coupling reactions (17-20).

Penicillamine (PEN) (Figure 1) is used in heavy metal poisoning, hepatitis and scleroderm. Several methods are reported for spectrophotometric determination using chromogenic reagents (21-22), its reducing action (23-25), employing charge transfer complexation (26) or difference spectrophotometry (27). Fluorimetric methods (28-29), polarographic and voltammetric (30, 31) and chromatographic methods (32-34) were also reported.

Viewing the reported spectrophotometric methods for determining CAP and PEN, revealed that the use of ligand exchange reactions using ternary or binary complexes was hitherto not reported.

The interactions of CAP and PEN with Ag(I) - Bromopyrogallol red - phenanthroline, or Copper (II) oxinate (CuQ_2_) chloroformic solution were developed into two spectrophotometric methods for their determination in their pure state and in their pharmaceutical preparations. The proposed methods proved to be simple and of accuracy and precision that are comparable to previously published methods, and selective for the two thiol containing drugs in presence of their disulphide oxidation products and common excipients of their pharmaceutical preparations.

## EXPERIMENTAL

### Instrumentation

The instrument used was Perkin-Elmer Lambda EZ201 UV-visible spectrophotometer with matched 1 cm quartz cells.

### Reagents and solutions

Drug solutions: Stock solution of each drug was prepared in distilled water to give 0.5 mg mL^-1^ and 40 μg mL^-1^ for CAP and PEN (Sigma Chem Co, Milwaukee-WI, USA), respectively for use in method I. Standard solutions containing 0.5 mg mL^-1^ and 40 μg mL^-1^ for PEN and CAP, respectively were prepared for use in method II.

**Reagents for Method I.**

- Bromopyrogallol red (BPR) solution, prepared by dissolving 250 mg (BPR) (Sigma Chem Co, Milwaukee-WI, USA) in 100 ml of 1% ammonium acetate.
- 1,10 phenonthroline (Phen) solution, 0.2% prepared by dissolving 200.0 mg of (Phen) (Aldrich Chem Co., Milwaukee-WI, USA) in 100 mL ethanol.
- Gelatin solution prepared by dissolving 500 mg gelatin (Sanofi Co, Paris, France) in 100 mL of warm distilled water.
- Silver nitrate solution was prepared to contain 25 μg mL^-1^ (protected from light).
- Ammonium acetate 20% w/v solution in distilled water.

**Reagents: for Method (II).**

- Copper quinolin-8-olate (CuQ_2_) (prepared by the gravimetric method (35)), by dissolving 60 mg of the solid reagent in 1 L of chloroform (Adjust the concentration to exhibit absorbance of about 1.0 at λ_max_ of 410 nm.
- Acetate buffer solutions of pH5-8.

### General procedure and construction of calibration graphs

**Method I.** Volumes of 0.2-1.1 mL of the standard solution were transferred to a set of 25 mL volumetric flasks, and the volume was completed to 1.1 mL with distilled water. Volumes of 1 mL 20% ammonium acetate, 0.5 mL gelatin solution, 0.5 mL of Phen, 2 mL of silver nitrate and finally 1 mL BPR were added to each flask. Solutions were mixed, completed to volume with distilled water and allowed to stand for 5 minutes at room temperature. A blank experiment was carried out by omitting CAP or PEN. Absorbance of the blank solution was measured against the sample solution at λ_max_ of 635 nm.

**Application to CAP tablets.** Twenty tablets were powdered and a quantity of the powder equivalent to 25 mg CAP were extracted with 25 mL of distilled water in a 50 mL volumetric flask and completed to volume with distilled water. The suspension was filtered and an aliquot volume of the filtrate was used for analysis as described under Method I.

**Application to PEN capsules.** The contents of 20 capsules were mixed and a weighed quantity of the mixed contents equivalent to 4 mg of PEN was transferred into a 100 mL volumetric flask containing 30 mL distilled water. The mixture was shaken mechanically for 10 minutes, completed to volume with distilled water, filtered and an aliquot volume of the filtrate was used for analysis as described under Method I.

**Method II.** Into 60 mL separating funnel, accurate volumes of standard solutions in the concentration range of 30-100 μg mL^-1^ for CAP and PEN were measured. The volumes were completed to 5 mL with distilled water and the pH of the solutions adjusted to pH 6 using pH paper. A volume of 10 mL of copper quinolin-8-olate solution was added and the mixture was shaken for 5 minutes with 10 mL of chloroform. The organic solvent layer was separated and dried over anhydrous sodium sulphate and the absorbance of the blank solution was measured against the sample at λ_max_ of 410 nm.

**Procedure for tablets and capsules.** Twenty tablets were weighted and powder or the contents of 20 capsules were mixed, and an accurately weighed quantity equivalent to 25 mg CAP or PEN was transferred into 50 mL volumetric flask half filled with distilled water, shaken for 15 minutes and diluted with the same solvent. The solution was filtered and first portion was discarded. Volumes of the filtrate were transferred into a series of 60 mL separators funnels and completed as described under Method II.

## RESULTS AND DISCUSSION

### Method I

**Figure 2:**
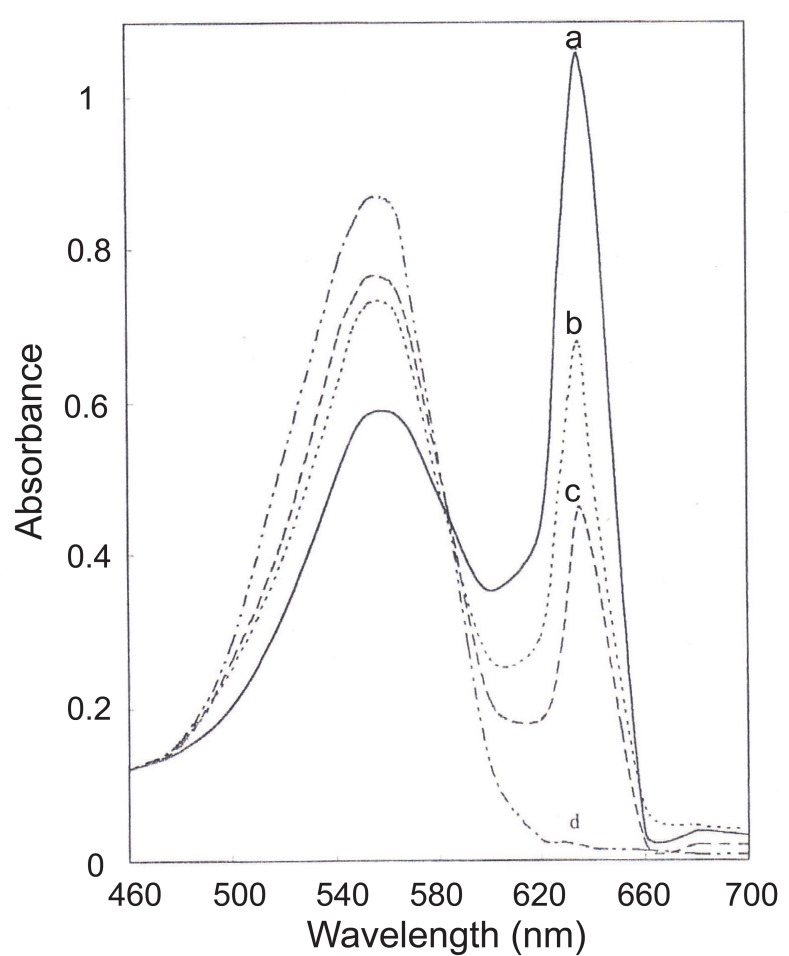
Absorption spectra of: (a) The ternary complex of [Ag-phen-BPR] solution, (b) In presence of 5 μg mL^-1^ CAP, (c) In presence of 1.6 μg mL^-1^ PEN, and (d) Absorption spectrum of (BPR) and (Phen) solution.

The ternary complex ([Ag(I) (Phen)]_2_ BPR) was first reported by Dagnal & West (36). We found that when CAP or PEN was added to this complex, a silver mercaptide of the drug is formed, and the colour of the ternary complex is decreased proportionally. Figure 2 illustrates the absorption spectra of the ternary complex in presence and absence of the investigated drugs at the same λ_max_ where the addition of the drugs caused only decrease in absorbance.

### Optimum reaction conditions

**Figure 3:**
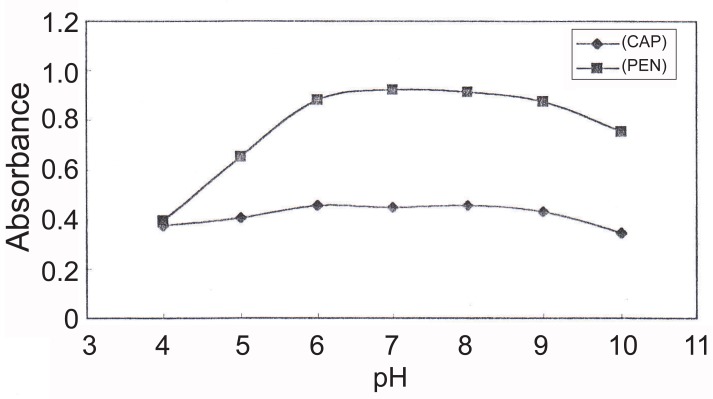
Effect of pH on the reaction of (Ag-phen-BPR) complex with 5 and 1.6 μg mL^-1^ of CAP and PEN, respectively.

**Figure 4:**
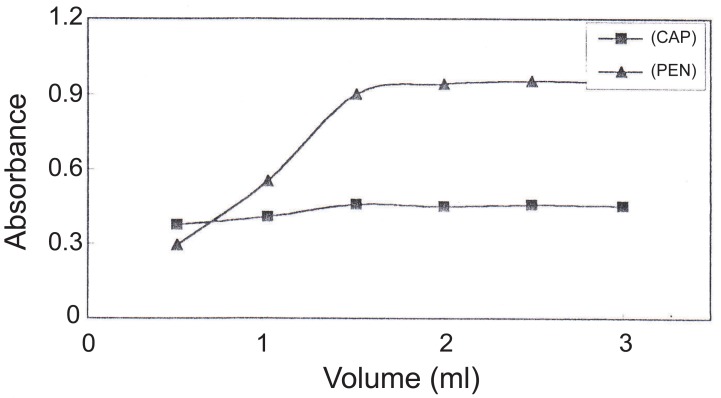
Effect of volume of silver nitrate 25 μg mL^-1^ on its reaction with 5 and 1.6 μg mL^-1^ of CAP and PEN, respectively.

- The effect of pH was studied over pH range of 4-10. The best results were obtained in the range of pH 6 - 8 using a solution of 2% w/v solution of ammonium acetate (Figure 3).
- The effect of reagent concentration: The ternary complex formation was optimized by using at least 4-fold molar excess of BPR and Phen, respectively over Ag(I). Two and half mL of 25 μg mL^-1^ of silver nitrate were suitable for CAP and PEN (Figure 4).
- Selection of stabilizing agent to avoid precipitation of silver ternary complex. Among Tritox X-100, sodium lauryl sulphate, gelatin solutions, a 0.5% gelatin solution was the best stabilizer which acts as a protective colloid.
- The stoichiometry of the reaction: The composition of the ternary complex was reported to be [Ag(I) (Phen)_2_, BPR] and its reaction with CAP and PEN as determined using continuous variation method (37) was found to be 1:1 (Drug to ternary complex).

### Calibration curves and statistical analysis

Table 1 shows the range of linearity and statistical analysis at λ_max_ of 635 nm on using method I. Table 2 illustrates accuracy and precision. The small RSD% and SAE indicate high precision and accuracy.

**Table 1:** Optical characteristics and statistical data of the regression equations for Captopril and Pencillamine reactions with (Ag(I)-phen-BPR) ternary complex (Method I)

Item	(CAP)	(PEN)

Beer’s law range μg mL^-1^	2-10	0.48-1.76
Sandell’s sensitivity μg mL^-1^ per 0.001 A	1.01 × 10^-2^	1.89 × 10^-3^
Regression equation (A):		
Intercept (a)	4.44 × 10^-2^	-3.90 × 10^-2^
t S_a_	6.88 × 10^-3^	3.52 × 10^-2^
Angular coefficient (b)	8.33 × 10^-2^	0.573
t S_b_	1.10 × 10^-3^	3.96 × 10^-2^
Correlation coefficient (r)	0.9999	0.9985
Linearity (S_b rel (%)_)	0.512	2.823
Variance (S_o_^2^)	6.360 × 10^-6^	2.06 × 10^-4^
Detection limit μg mL^-1^	7.10 × 10^-2^	5.67 × 10^-2^

**Table 2:** Evaluation of accuracy and precision for Method I

Drug	μg mL^-1^ added	Mean found	SD	RSD%	SAE

CAP	4	4.02	0.095	2.63	0.042
	5	5.07	0.108	2.13	0.048
	6	6.00	0.056	0.93	0.025
	Overall		0.086	2.14	0.038
PEN	0.8	0.787	0.033	4.193	0.015
	1.12	1.11	0.018	1.619	0.008
	1.44	1.41	0.064	4.53	0.029
	Overall		0.038	3.44	0.017

SD, Standard deviation; RSD, Relative standard deviation; SAE, Standard analytical error.

Application to tablets and capsules: The results of applying method I to analyse CAP and PEN in dosage forms and comparison with reference methods as shown in Table 3 indicates that fairly comparable results of developed method with reference methods (38) by applying statistical analysis.

**Table 3:** Determination of CAP and PEN in their preparation by Method I

Drug	Number of experiments	Proposed mean%	Method CV%	Reference Mean%	Method of (38) CV%

Captopril					
Capoten Tablets (25 mg)	5	100.02	0.532	100.69	0.905
Facopril Tablets (25 mg)	5	97.70	0.705	98.66	0.984
Lotensine Tablets (25 mg)	5	99.53	1.06	98.99	0.526
Captopril Tablets (25 mg)	5	101.23	1.21	98.99	0.52
Pencillamine Artamin Capsules (100 mg)	5	100.45	1.11	101.3	1.05

CV, Coefficient of variation.

### Method II

**Figure 5:**
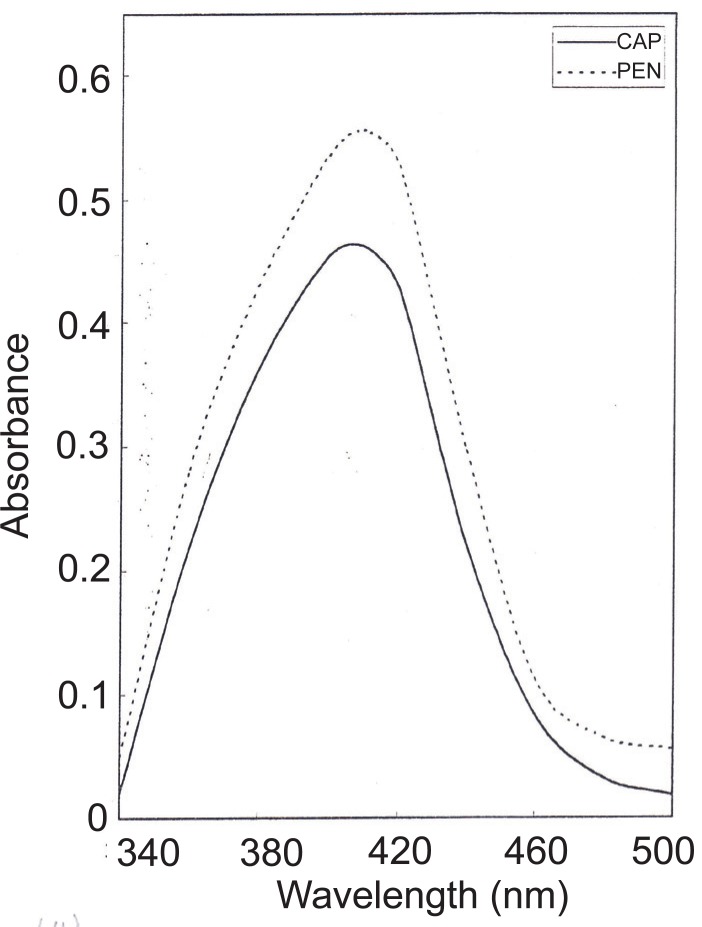
Absorption spectra of copper quinoline-8-olate (60 mg L^-1^) in presence of 50 and 60 μg mL^-1^ of CAP and PEN, respectively.

8-hydroxyquinoline (oxine) forms coloured chelates with metal ions including copper (II) (39) which are extractable with chloroform (CuQ_2_). In this method we found that when the chloroformic Cu(II)-oxine solutions was shaken with CAP or PEN, a decrease in absorbance of the chelate at λ_max_ of 410 nm occurred which was proportional to the drug added. Figure 5 shows the absorption spectrum of CuQ_2_ in presence of CAP or PEN.

For CAP, it may be assumed that it reduces Cu(II) to Cu(I) and CAP itself is oxidized to its disulphide (40). For PEN, a coupled redox complexation reaction takes place in which the disulphide formed gives Cu(I)-PEN chelate (41), and a decrease in absorbance of CuQ_2_ happens.

2 RSH (CAP) + 2 CuQ_2_ → R SSR + 2 Cu^+^ + 2Q (free oxine)

4 RSH (PEN) + 2 CuQ_2_ → RSSR + 2RSCu^+^ + 4Q (free oxine)

**Figure 6:**
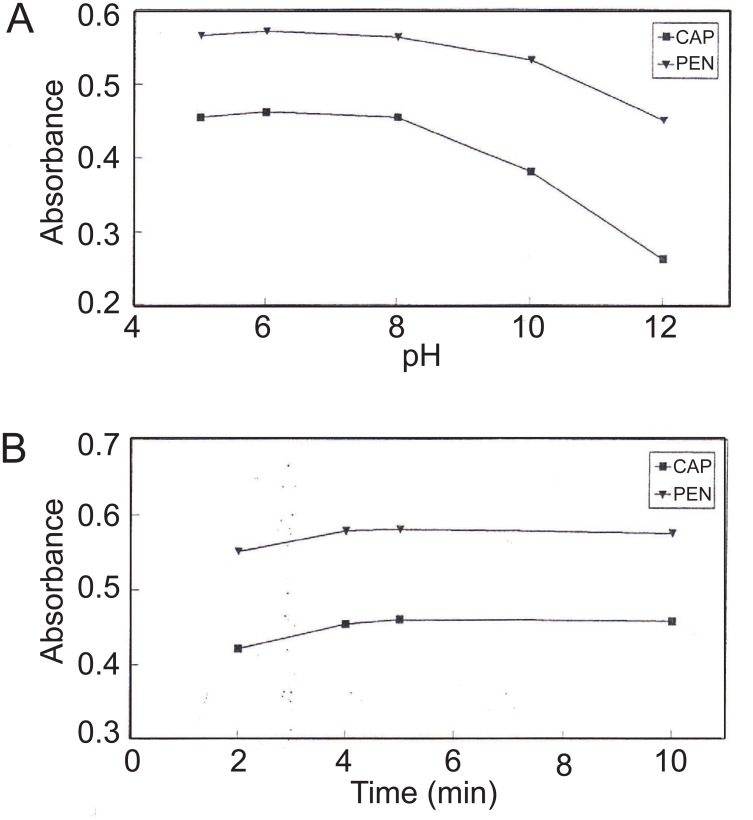
A, Effect of pH of the aqueous phase on decoloration of chloroform solution of copper quinoline-8-olate in presence of 50 and 60 μg mL^-1^ of CAP and PEN, respectively; B, Effect of shaking time on decoloration of chloroform solution of copper quinoline-8-olate in presence of 50 and 60 μg mL^-1^ of CAP and PEN, respectively.

Both CAP and PEN produce a proportional decrease in absorbance of CuQ_2_ in chloroform. The optimum conditions for the reaction were studied in the optimum pH 8 buffer solution (Figures 6a, 6b), shaking time for 5 minutes, and optimum ratio of aqueous to organic layer of 1:2 (aqueous to chloroformic).

Calibration curves and statistical analysis of results of the use of method II, the linearity range, correlation coefficients and variances are shown in Table 4.

**Table 4:** Optical characteristics and statistical data of the regression equations for Captopril and Pencillamine reactions with copper quinolin-8-olate (Method II)

Item	(CAP)	(PEN)

Beer’s law range μg mL^-1^	30-90	30-100
Sandell’s sensitivity μg mL^-1^ per 0.001 A	0.107	0.109
Regression equation (A):		
Intercept (a)	-2.63 × 10^-2^	-3.38 × 10^-2^
t S_a_	1.22 × 10^-2^	2.03 × 10^-2^
Angular coefficient (b)	9.85 × 10^-3^	9.84 × 10^-3^
t S_b_	1.92 × 10^-4^	3.03 × 10^-4^
Correlation coefficient (r)	0.9999	0.9996
Linearity (S_b rel (%)_)	0.760	1.197
Variance (S_o_^2^)	1.57 × 10^-5^	5.47 × 10^-5^
Detection limit μg mL^-1^	0.944	1.763

Accuracy and precision are illustrated in Table 5 indicating high precision and accuracy (low RSD% and SAE).

**Table 5:** Evaluation of accuracy and precision for Method II

Drug	Number of experiments	μg mL^-1^	Mean found%	SD	SAE

Captopril	5	40	98.59	0.963	0.431
	5	50	99.28	1.10	0.496
	5	70	100.31	0.914	0.409
Pencillamine	5	40	99.36	0.986	0.993
	5	60	100.45	1.139	0.505
	5	80	99.98	1.159	0.518

SD, Standard deviation; SAE, Standard analytical error.

The proposed method (II) was applied to both CAP and PEN in dosage forms and was compared with a reference method in Table 6. Comparable results obtained by both methods by students’ t test and variance ratio F test.

**Table 6:** Determination of CAP and PEN in their preparation by Method II

Drug	Number of experiments	Proposed Method Mean%	CV%	Reference Method (38) Mean%	CV%

CAP					
Capoten Tablets (25 mg)	5	99.96	0.884	100.69	0.905
Farcopril Tablets (25 mg)	5	98.15	0.454	98.66	0.985
Lotensine Tablets (25 mg)	5	99.44	0.428	98.99	0.526
Captopril Tablets (25 mg)	5	100.23	1.011	101.69	1.258
PEN Artamin Capsules (100 mg)	5	100.86	0.630	101.30	1.058

CV, Coefficient of variation.

### Interference study in methods I and II

It was found that common tablets and capsules excipients as well as oxidized drugs (disulphide) did not interfere in both procedures. Hydrochlorothiazide (commonly prescribed with CAP) is insoluble in water; consequently it does not interfere. The proposed procedures are recommended as simple method in quality control laboratories for the analysis of CAP or PEN in the pure state and dosage forms.

### Comparison between methods I and II

Method I is of higher sensitivity than method II. Regarding accuracy and precision, both methods are equivalent, and results of both methods are comparable to the reference spectrophotometric methods applied simultaneously. Method I is relatively advantageous to method II because of the ease by which solutions were prepared.

## CONCLUSION

Two simple spectrophotometric methods based on application of ligand exchange reactions were presented. The described methods were applied on two thiol group containing compounds: captopril and penicillamine. No interference could be observed from the disulphide degradation products of the two compounds. The applicability of the developed methods was evaluated through the determination of the two drugs in bulk form and in several pharmaceutical formulations with good accuracy and precision.
